# Anxiety level and correlates in methamphetamine-dependent patients during acute withdrawal

**DOI:** 10.1097/MD.0000000000006434

**Published:** 2017-04-14

**Authors:** Hang Su, Jie Zhang, Wenwei Ren, Ying Xie, Jingyan Tao, Xiangyang Zhang, Jincai He

**Affiliations:** a Department of Neurology, The First Affiliated Hospital of Wenzhou Medical University, Wenzhou; b Shanghai Mental Health Center, Shanghai Jiaotong University School of Medicine; c Department of Neurology, Zhongshan Hospital, Fudan University, Shanghai; d Department of Neurology, The First Affiliated Hospital of Yangtze University, Jingzhou; e Sir Run Run Shaw Hospital, School of Medicine, Zhejiang University, Hangzhou; f Beijing HuiLongGuan Hospital, Peking University, Beijing, China; g Department of Psychiatry and Behavioral Sciences, Harris County Psychiatric Center, The University of Texas Health Science Center at Houston, Houston, TX.

**Keywords:** abstinence, anxiety symptoms, methamphetamine, prevalence, risk factors

## Abstract

Anxiety is often a core element of withdrawal symptoms; however, risk factors associated with anxiety symptoms during the early stage of withdrawal in methamphetamine (METH) users are not well understood. Two hundred ten METH-dependent subjects who had been abstinent for 1 to 7 days were recruited. We used a set of self-administrative questionnaires eliciting information on sociodemographics, detailed drug use history and anxiety. Beck Anxiety Inventory (BAI) was used to measure anxiety symptoms. METH users had a mean BAI score of 6.9; 72 (34.3%) of the study sample had anxiety symptoms during acute METH withdrawal, including 42 (20.0%) with mild anxiety, 25 (11.9%) with moderate anxiety, and 5 (2.4%) with severe anxiety. In addition, gender (female), higher frequency of drug use, and history of polysubstance use were significantly correlated with anxiety symptoms during acute METH withdrawal. Anxiety symptoms appear to be common during the first week of METH abstinence, and several risk factors are identified.

## 1 Introduction

Methamphetamine (METH), a highly addictive and potent psychostimulant, is one of the most widely abused illicit drugs in the world.^[1]^ Chronic METH exposure produces a variety of adverse effects, including cognitive deficits, neurotoxicity, and even psychosis episodes.^[2–4]^ An abrupt discontinuance of chronic METH exposure results in withdrawal symptoms, mainly consisting of depression, anxiety, disturbed sleep, and craving.^[5–7]^

Consistent evidence showed that chronic METH exposure can lead to negative emotional states. In rats, chronic METH administration increases anxiety-like behaviors during METH withdrawal.^[8]^ In addition, 1 study showed that rats exhibit anxiety-like behaviors when measured at both 24 hours and 2 weeks of amphetamine withdrawal.^[9]^ Intriguingly, yohimbine, an anxiogenic drug, could induce reinstatement of METH seeking in a rat relapse model and cocaine seeking in squirrel monkeys,^[10,11]^ supporting the notion that negative emotional states lead to drug seeking and relapse.^[12]^

In clinical study, anxiety is one of the most common comorbid condition in METH users.^[13,14]^ Several studies showed that 76% of subjects reported anxiety symptoms after the onset of amphetamine use and 39% of amphetamine users reported a history of anxiety disorders.^[15,16]^ Likewise, an Australian research demonstrated that nearly 40% of METH treatment entrants reported a history of anxiety disorders,^[17]^ which were associated with negative drug-use outcomes.^[18]^ Furthermore, anxiety is one of the most prominent symptoms of METH withdrawal during the first several weeks of abstinence.^[5,7]^ Importantly, failure to manage METH withdrawal symptoms may contribute to relapse.^[19]^

The mechanism of anxiety in METH abstinence is manifold. The hippocampus plays an important role, and serotonin (5-HT), dopamine, and related receptors are involved in this process. For example, increasing 5-HT levels in the ventral hippocampus could reverse anxiety-like behavior induced by amphetamine withdrawal in rats.^[20]^ Anxiety also increased the expression of gamma-aminobutyricacid A (GABAA) receptors during METH withdrawal.^[21]^ Furthermore, the expression and activity of corticotropin releasing factor type 2 receptors in the dorsal raphe nucleus modulate anxiety behaviors observed during amphetamine withdrawal.^[22]^

From the above, identifying risk factors that predict anxiety symptoms during acute METH withdrawal may facilitate the development of new prevention and treatment strategies for addiction and relapse. To date, the level and risk factors of anxiety symptoms during acute METH withdrawal were not well understood. In this study, our aim was to explore the potential correlates of anxiety symptoms during acute METH withdrawal.

## 2 Methods

### 2.1 Study design

This is an observational study according to its cross-sectional design.

### 2.2 Subjects and setting

A cross-sectional study was conducted from November 2012 to June 2013 at Wenzhou Sanyang Detoxification Institute, which is located in Wenzhou City, Zhejiang province, China. Inpatients have no access to METH and other illegal drugs in the institute, which allows for strict control of abstinence. Two hundred ten subjects were recruited in this study. Subjects were included in the study based on the following criteria: be between 18 and 50 years of age; have had a positive urine test when admitted to the institute; meet Diagnostic and Statistical Manual of Mental Disorders-IV (DSM-IV) criteria for METH dependence; have been abstinent for 1 to 7 days; and signed informed consent. The subjects were excluded if they were seropositive for Human Immunodeficiency Virus, or met DSM-IV criteria for axis I psychiatric disorders, or had significant physical illnesses such as stroke or cardiovascular diseases. Since all the subjects are local residents, it has good representation for METH-dependent patients in southeast coastal region of China.

The study protocol was approved by the Human Research and Ethics Committee of Wenzhou Medical University. After detailed explanations of this study, written informed consents were obtained from all subjects.

### 2.3 Measures

A retrospective chart review of drug-related and sociodemographic characteristics of all METH-dependent patients was conducted. Each participant was interviewed and completed a detailed case report form that recorded general information, sociodemographic characteristics, drug-use-related information, cigarette smoking, alcohol drinking, and anxiety symptoms. Each Subject completed the Beck Anxiety Inventory (BAI).^[23]^ The BAI is a 21-item self-report inventory for measuring severity of clinical anxiety. Each item is scored from 0 to 3 with cumulative scores ranging from 0 to 63. The score of 0 to 7, 8 to 15, 16 to 25, and 26 to 63 were classified as no anxiety, mild, moderate, and severe anxiety, respectively.

### 2.4 Statistics analysis

Descriptive statistics was used to summarize the characteristics of the study sample including social–demographic characteristics, drug-use-related information, cigarette smoking, alcohol use history, BAI score, and prevalence of anxiety symptoms. The analysis of social–demographic characteristics, drug-use-related information, cigarette smoking, and alcohol use history with different anxiety status was conducted using logistic regression and Pearson chi-square test. A multivariate logistic regression model was constructed using a forward likelihood ratio sequence. All variables that were significantly associated with anxiety status in the univariate logistic regression were then entered in a multiple logistic regression controlling for the potential effects of gender, current age, and education level. All analyses were performed using Statistical Product and Service Solutions software (SPSS, Inc., Chicago, IL). A 2-tailed *P* value of less than 0.05 was considered to be statistically significant.

## 3 Results

### 3.1 Characteristics and pattern of drug use

A total of 210 METH-dependent inpatients during acute withdrawal (1–7 days from last drug use) were recruited in this study. Characteristics of the study sample including social-demographics, drug use history, cigarette smoking history, and alcohol drinking history are summarized in Table 1. The ages of the study sample ranged from 18 to 50 years (mean, 31.33 years), and 101 subjects (48.1%) were aged less than 31 years. The majority (81%) of subjects were male, 129 (61.4%) had junior high school or less education, 105 (50.0%) were unmarried, 77 (36.7%) were unemployed, 85 (40.5%) lived in rural area, 11 (5.2%) reported a family history of mental illness. All participants were Han Chinese people.

**Table 1:**
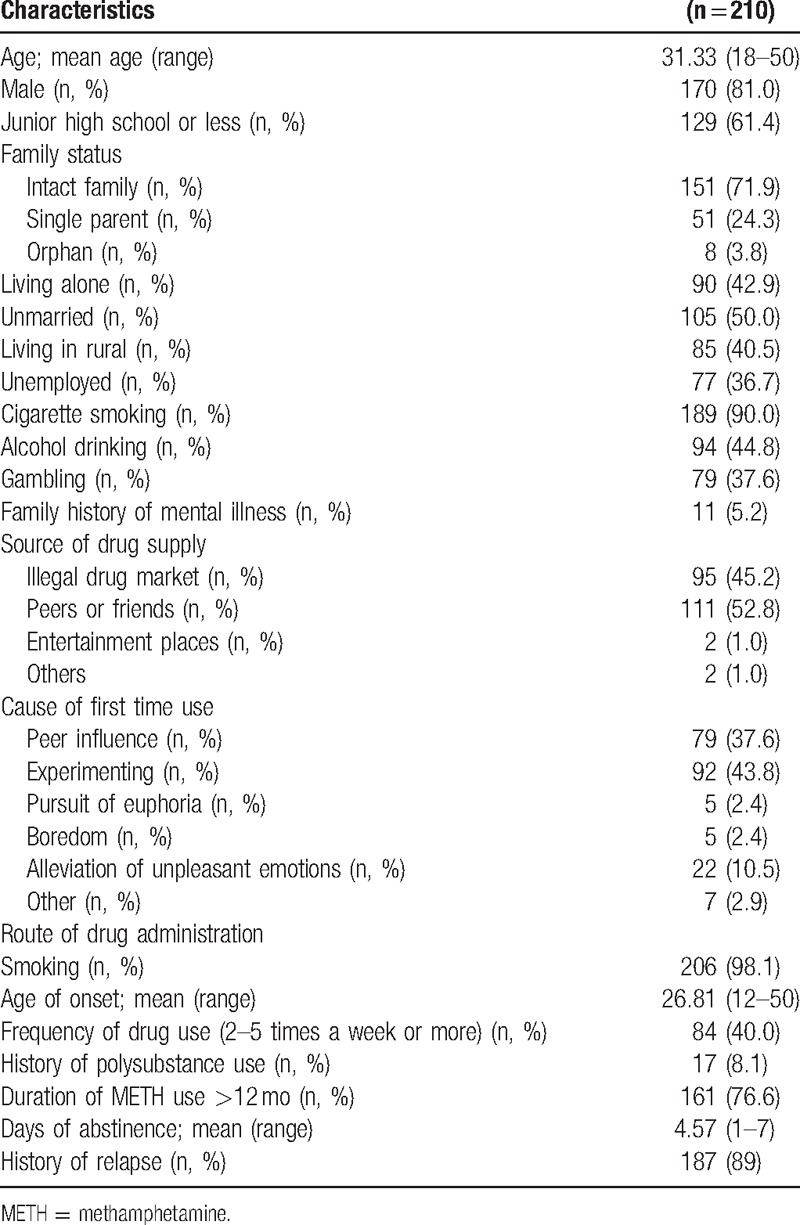
Characteristics of the study group.

Among 210 METH users during acute withdrawal, the average days of abstinence were 4.57 days ranging from 1 to 7 days. The onset age of METH use ranged from 12 to 50 years (mean, 26.81 years). The majority (98.1%) of study sample used METH by smoking, 84 (40.0%) used METH 2 to 5 times a week or more, 161 (76.6%) had used METH for more than 1 year, 187 (89.0%) reported a history of relapse. Seventeen subjects (8.1%) reported a history of polysubstance use, indicating the use of 2 or more drugs, including heroin, ketamine, ecstasy, and METH. The main causes of drug use for the first time included experimenting (43.8%), peer influence (37.6%), pursuit of euphoria (2.4%), boredom (2.4%), alleviation of unpleasant emotions (10.5%), and others (2.9%). The main drug resources included supply from peers or friends (52.8%), illegal drug market (45.2%), entertainment places (1.0%), and others (1.0%).

### 3.2 Anxiety symptoms and associated factors during METH withdrawal

METH users had a mean BAI score of 6.9; 72 (34.3%) of the study sample had anxiety symptoms during acute METH withdrawal, including 42 (20.0%) with mild anxiety, 25 (11.9%) with moderate anxiety, and 5 (2.4%) with severe anxiety. The scores of each item of BAI listed in Table 2. The analysis of univariate logistic regression demonstrated that anxiety symptoms (BAI ≥ 8) were significantly related with 3 factors listed in Table 3, including gender, frequency of drug use, and history of polysubstance use. Then these factors as well as general information including current age and education level were entered in the multivariate logistic regression model. The multiple logistic regression showed that gender (female), higher frequency of drug use (2–5 times a week or more), and history of polysubstance use were associated with anxiety symptoms during acute METH withdrawal, and the adjusted odds ratios (OR) were 2.6 (95% confidence interval [CI]: 1.28–5.48), 1.84 (95% CI: 1.00–3.84), and 3.86 (95% CI: 1.33–11.25), respectively (Table 4).

**Table 2:**
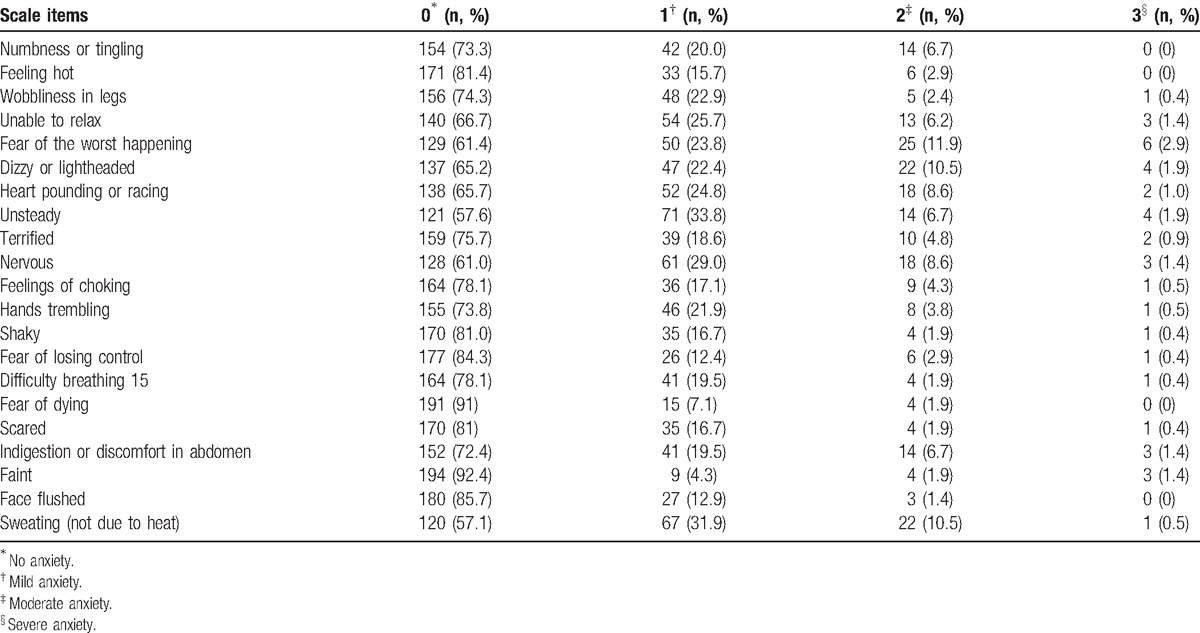
The values for each of the items in the BAI (n = 210).

**Table 3:**
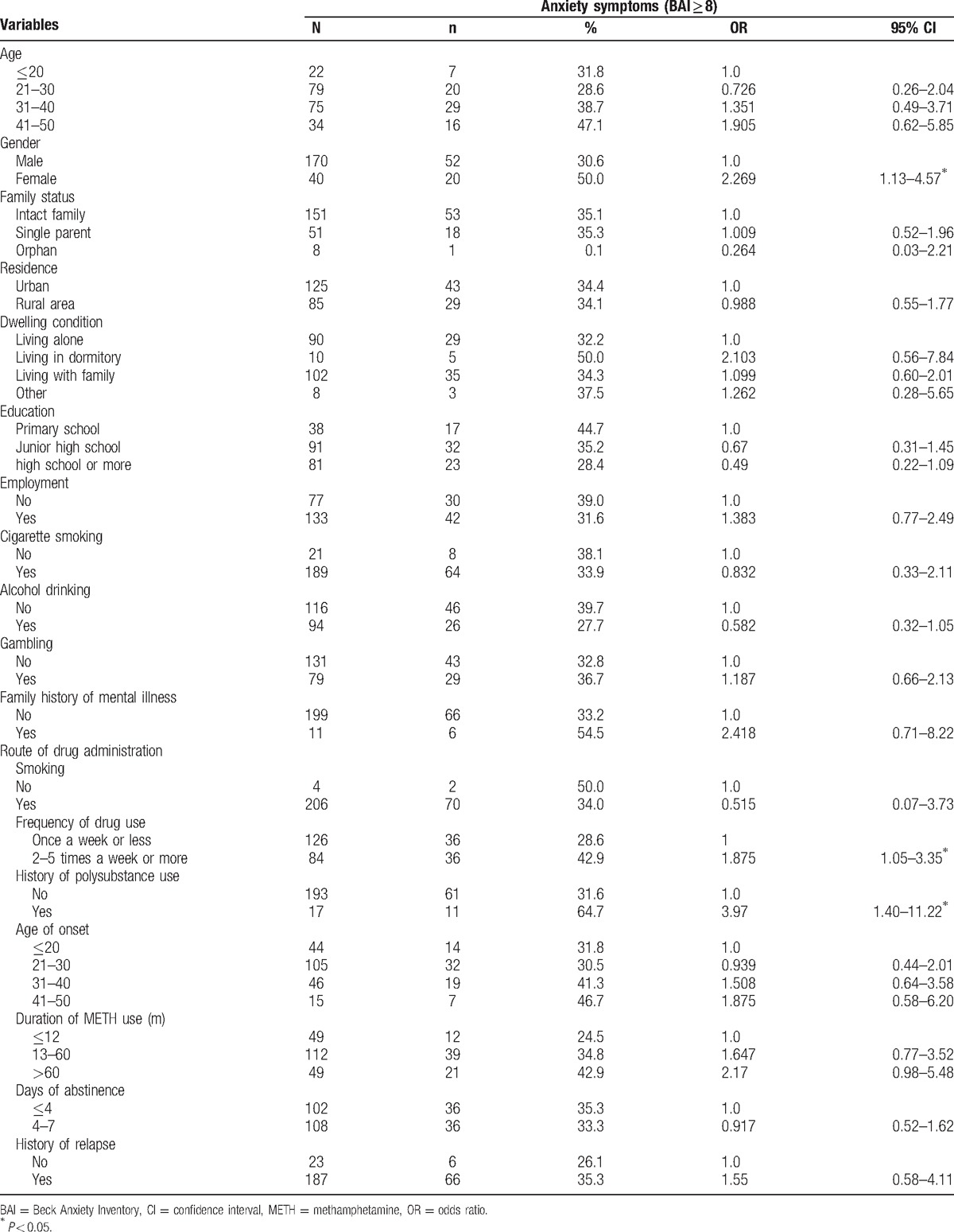
Bivariate analysis of risk factors for anxiety symptoms in METH acute withdrawal.

**Table 4:**
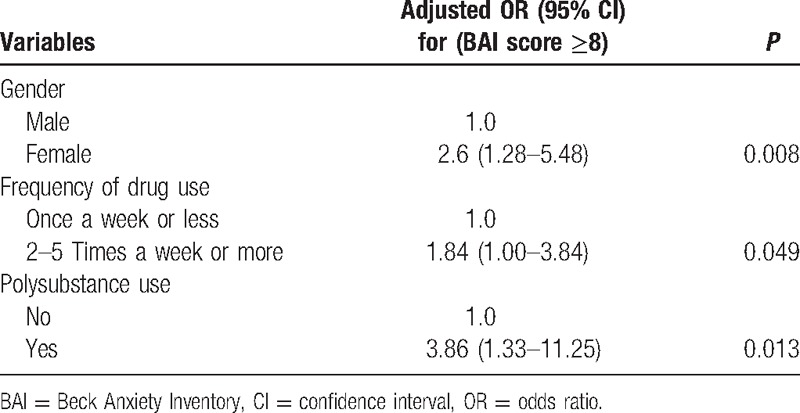
Multivariate logistic regression of risk factors for anxiety symptoms during METH early withdrawal.

## 4 Discussion

To date, this is the first study to investigate the relationships between risk factors and anxiety symptoms among METH-dependent patients during acute METH withdrawal. In addition, our study reported a rate (34.3%) of anxiety symptoms in METH users during the first week of abstinence. The findings of our study might contribute to a better understanding of the negative emotional state and the development of new measures to prevent and treat anxiety during acute METH withdrawal.

Our study demonstrated that anxiety symptoms were common during METH withdrawal and the rate of anxiety symptoms was high (34.3%) (4.0% in normal population), including 20.0% with mild anxiety, 11.9% with moderate anxiety, and 2.4% with severe anxiety according to BAI score. These results were consistent with previous studies. For instance, an anxiety-like behavior (i.e., increased latency to enter open arms on the elevated plus maze) was observed in rats at both 24 hours and 2 weeks of amphetamine withdrawal.^[9]^ In addition, 1 study showed that this anxiety state persisted for 4 weeks after cessation of amphetamine administration in rats.^[24]^ Likewise, in humans, anxiety is one of the most common symptoms of METH withdrawal during the first several weeks of abstinence and may fade away thereafter.^[5,7]^ Taken together, the aforementioned studies suggested a notion that anxiety symptoms are major elements of METH withdrawal syndrome.

The multiple logistic regression analysis demonstrated that gender (female), frequency of drug use, and history of polysubstance use were associated with anxiety symptoms (BAI ≥ 8) during acute METH withdrawal. In present study, we found that females had a higher risk of anxiety symptoms during acute METH withdrawal (OR = 2.6, 95% CI = 1.28–5.48, *P* = 0.008). Several lines of previous work are consistent with this finding. For example, researches in nearly all anxiety disorders, including obsessive compulsive disorder,^[25]^ generalized anxiety disorder,^[26]^ panic disorder,^[27]^ social anxiety disorder,^[28]^ and posttraumatic stress disorder,^[29]^ have consistently demonstrated that prevalence rates were higher in women than men. The underlying mechanisms for gender differences in prevalence rats were not well understood. However, a variety of relevant factors, like biological influences, social and environmental factors and trauma, and so on, have been suggested to explain this gender difference.^[30]^ Nevertheless, in our study, the relatively small sample size of women is a drawback. Therefore, in order to increase the statistic power, further study with more women subjects will be needed.

In the present study, we also found that METH users with higher frequency of drug use (2–5 times per week or more) experienced more anxiety symptoms than those with lower frequency of drug use (once per week or less) during acute METH withdrawal. This finding may be partly explained by the fact that increased drug use frequency is commonly used as a means of coping with considerably negative emotional state after the discontinuance of chronic drug use.^[31]^ For example, preclinical studies showed that drug withdrawal after long-term METH administration could lead to negative emotional-like state,^[32]^ which was highly related with the increase of drug intake.^[33]^ Nevertheless, in humans, whether higher frequency of drug use, which is related with high dose of drug use,^[34]^ contributed to more serious anxiety symptoms during early abstinence remains unclear due to the cross-sectional design. In all, future longitudinal studies will be needed to elucidate the causal relationship between frequency of drug use and anxiety symptoms.

In addition, our study also showed that those who reported a history of polysubstance use were more likely to experience anxiety symptoms during early METH withdrawal. In our study, 17 METH users reported a history of polysubstance use, indicating the use of 2 or more drugs, including heroin, ketamine, ecstasy, and other illegal drugs during chronic METH exposure. This result seemed consistent with previous studies. For example, increased consumption of other drugs, including cannabis, temazepam, opiates, diazepam, and so on, was reported as a common means of coping with negative emotional states during amphetamine withdrawal.^[35]^ More recently, a 10-year prospective study showed that polydrug use was associated with mental distress, including anxiety and depression.^[36]^ In present study, we cannot give answers to the causality of the relationships between polydrug use and anxiety symptoms. Moreover, the comparatively small sample of those with a history of polydrug use is a concern, which constrained the interpretation of the results.

Several limitations of our study should be addressed. Because of the study design and lack of comparison, it is not possible to find that underlying conditions like gender, duration of misuse, dose, age are responsible for differences or the withdrawal, we could only make a conclusion that these factors are correlated with anxiety during acute METH withdrawal. Second, due to the cross-sectional design, we could not confirm the causality of the relationships between risk factors and anxiety symptoms during early METH withdrawal and longitudinal studies are needed. Besides, this was a retrospective study; therefore, observer and reporting bias could not be ruled out. Furthermore, the present investigation was conducted at the compulsory detoxification institute and may not generalize our findings to the larger METH-using community. Finally, we did not apply rating scales or structured instruments for comorbidity evaluations of personality disorder, psychiatric symptoms and suicidal behavior, and so on.

In summary, the current investigation demonstrated that METH users had a high prevalence of anxiety symptoms during early METH withdrawal. In addition, anxiety symptoms during early withdrawal were associated with several risk factors, including gender (female), frequency of drug use, and history of polysubstance use. A better understanding of prevalence and risk factors of anxiety symptoms during withdrawal might facilitate the development of new prevention and treatment strategies for addiction and relapse.
